# Prostate Cancer Screening Trends After United States Preventative Services Task Force Guidelines in an Underserved Population

**DOI:** 10.1089/heq.2018.0004

**Published:** 2018-05-01

**Authors:** Neel H. Patel, Jonathan Bloom, Joel Hillelsohn, Sean Fullerton, Denton Allman, Gerald Matthews, Majid Eshghi, John L. Phillips

**Affiliations:** ^1^ Department of Urology, New York Medical College, Valhalla, New York.; ^2^ Urologic Oncology Branch, National Cancer Institute, Bethesda, Maryland.

**Keywords:** prostate biopsy, prostate cancer, Prostate Specific Antigen, urban health, USPSTF

## Abstract

**Purpose:** Prostate cancer screening is a controversial topic. We examined trends in Prostate Specific Antigen (PSA) testing in an underserved population before and after the United States Preventative Services Task Force (USPSTF) recommendation against screening.

**Methods:** Data were collected on all PSA and cholesterol screening tests from 2008 to 2014. We examined the trend of these tests and prostate biopsies while comparing this data to lipid panel data to adjust for changes in patient population.

**Results:** A decrease in PSA screening was observed from 2010 through 2014, with the greatest decline in 2012. The age group most affected was patients aged 55–69 years. The amount of prostate biopsies during this period decreased as well.

**Conclusions:** Decreased rates of PSA screening were observed in our urban hospital population that preceded the publication of the USPSTF guidelines. The incidence of prostate biopsies decreased in this timeframe. It now remains to be demonstrated whether decreased PSA screening rates impact the diagnosis of and ultimately the survival from prostate cancer.

## Introduction

Prostate cancer is the second leading cause of cancer-related death in men.^1^ Prostate Specific Antigen (PSA) screening offers improved detection rates of prostate cancer with the downside of overtreatment of low-risk disease.^2,3^ Treatment is often associated with significant side effects causing a change in quality of life related to urinary, sexual, and bowel function.^4^ Much of this morbidity also stems from the overtreatment of very-low and low-risk disease that may be better suited for active surveillance (AS).^5^ AS has gained in popularity but is not yet universally accepted by all who may benefit.^6^

Two large but controversial randomized trials assessed the impact of prostate cancer screening on mortality and became the basis for recommendations by the United States Preventative Services Task Force (USPSTF). The Prostate, Lung, Colorectal and Ovarian (PLCO) trial assigned over 75,000 men to annual PSA screening and a similar self-reported “control” group who did not undergo regimented PSA screening. PLCO reported no difference in cancer-specific mortality at 10 years between the two groups.^7^ The control group of the PLCO trial appears to have actually undergone equal or more intense PSA screening than the arm assigned to PSA testing.^8^

In contrast, the European Randomized Study of Screening for Prostate Cancer (ERSPC) evaluated over 160,000 men in multiple nations with different PSA screening approaches. The ERSPC demonstrated a 20% reduction in prostate cancer-specific mortality at a median follow-up of 9 years, but over 1400 patients needed to be screened to prevent one prostate cancer death.^9^ Based on the PLCO and ERSPC findings, the USPSTF concluded that the harm of PSA screening outweighed the benefits and assigned a grade D recommendation against screening in early 2012.^10^ After these guidelines were published, PSA screening remained controversial and individual screening practices have continued although highly varied.^11,12^ This is due to physicians, medical bodies and patients already established opinions regarding PSA screening.

After the USPSTF released this guideline, a survey of men aged 40–74 years was conducted to assess PSA awareness and men's responses to it.^11^ Overall, 62% of men agreed with the USPSTF grade D recommendation but only 13% intended to decline PSA testing, while 54% preferred to obtain a PSA test. The American Urological Association (AUA) guideline on early detection of prostate cancer does not advise screening in men younger than 40 or older than 70 years, or those with less than a 10-year life expectancy.^13^ The AUA and American Cancer Society (ACS) currently recommend that PSA testing be offered to asymptomatic men aged 55–69 years (AUA) or men older than 50 years with a minimum 10-year life expectancy (ACS) after patients receive information about the harms and benefits associated with screening.^14^

The AUA also reexamined their screening recommendations during this time period and downgraded their recommendation on PSA screening from an A to an overall C with the caveat that men aged 55–69 should undergo “shared decision making” with their physician concerning the potential harms and benefits of PSA screening.^15^ Currently, the USPTSF has now released a preliminary grade C recommendation in 2017.^16^ The reasoning behind the upgrade in recommendation was due to the ERSPC trial's reported continued benefit from screening in those aged 55–69, the mitigated risk of overtreatment with increased use of AS, and those most at risk for prostate cancer likely benefiting from screening, namely African Americans (AA) and men with a family history of disease. The result of the 2012 USPSTF recommendations is still being assessed in terms of changes in PSA screening intensity, and, ultimately, changes in prostate cancer specific-related death. Houston et al., found a decreased overall screening with a 7.5% reduction in the incidence of localized prostate cancer but a 1.4% increase in the incidence of metastatic disease.^17^

The risk of death from prostate cancer varies among different socioeconomic groups as higher socioeconomic status has been associated with easier access to screening and ultimately biopsy and diagnosis.^18^ Additionally, those without insurance, AA and Hispanic populations, and lower socioeconomic status more often present at a more advanced stage.^19^ While this may be due to a delay in diagnosis, there is some evidence that AA men may present with more aggressive disease as there is a higher rate of upgrading at the time of radical prostatectomy and higher rates of biochemical recurrence.^20,21^

In this study, we examined trends in PSA screening at an urban hospital in a designated medically underserved population with an increased incidence of prostate cancer of 21% compared with the general population.^22–24^

## Methods

### Patient data

After Internal Review Board approval, the number of PSA and, as a control, lipid panel tests were recorded for all patients by all providers, regardless of specialty, in a public, urban community hospital from January 2008 to December 2014. We used the ICD −9 code V76.44 or “screening for malignant neoplasms of prostate” to populate a de-identified database, excluding patients who received PSA for monitoring prostate cancer (ICD- 9 code 185). PSA screening tests were extracted from the health system central laboratory, including variables of month and year of service, PSA value, patient age, and race. To provide adjustments for changes in patient population and practice, we compared the ratio of number of PSA screening tests to lipid panel screening tests done for male patients at our institution. Furthermore, we stratified the data into age ranges commonly used for prostate cancer screening guidelines (<40, 40–54, 55–69, ≥70) and performed subset analyses. Insurance status were not available for these subjects, however, this hospital is located in a New York State Department of Health (DOH)-designated underserved area.

Prostate biopsy data from the same time period were collected and analyzed to further assess the effect of PSA screening on the use of diagnostic procedures.

### Statistics

PSA and lipid panel screening cohorts were analyzed using bivariate linear regression and subset analyses were performed to evaluate for differential changes in screening based on patient characteristics such as age and use of prostate biopsy. Patient characteristics were compared between the pre and post-USPSTF recommendation cohorts. We compared PSA data against lipid panel data to control for variations in population. Statistical testing was two-sided with a statistical significance set with an alpha of 0.05.

## Results

A total of 16,333 PSA screening tests were performed from January 2008 to December 2014 at the study institution that met the inclusion criteria. A monthly average of 194±92 PSA tests were performed compared to 148,610 lipid panel tests in these men with a monthly average of 2054±247. Table 1 shows the demographic data between between 2008 and 2014, where the mean age of patients who had PSA screening performed was 66.4 and 62.2 years respectively. The breakdown by race between 2008 and 2014 was 60% AA, 23% Hispanic, and 5% Caucasian versus 58% AA, 24% Hispanic, and 5% Caucasian, respectively.

**Table 1.** **Demographic Information for Study Population in 2008 and 2014**

	2008	2014	*p*
PSA screening (n)	3307	1234	—
Median age	66.4	62.2	<0.001
Race (%)			
African-American	60	58	
Hispanic	23	24	
Caucasian	5	5	
Asian	2	2	
Other	10	11	

PSA, Prostate Specific Antigen.

Figure 1 demonstrates the change in PSA screening at our institution over the study period. PSA screening by years shows that testing remained roughly stable from 2008 to 2009, but then began to decline in 2010 having the largest change in 2012 (β=−407.86, *p*=0.001) with a 29% decline. When analyzing these trends by month, the similar change was still appreciated with the lowest screening rates observed toward the end of the year (β=−3.01, *p*>0.001).

**FIG. 1.**
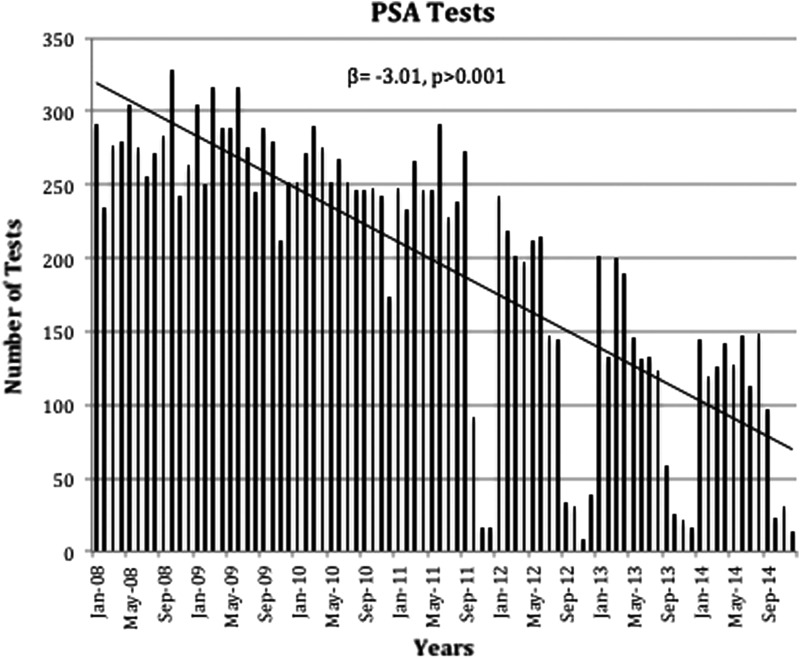
PSA tests ordered from 2008 to 2014 by months. PSA, Prostate Specific Antigen. β, slope; p, *p*-value.

As seen in Figure 2, we present the data for Lipid Panel testing showing that there were no substantial changes and screening remained relatively stable throughout the study years (β=37.89, *p*=0.673). Likewise, the trend by months shows similar changes (β=−0.11, *p*=0.922).

**FIG. 2.**
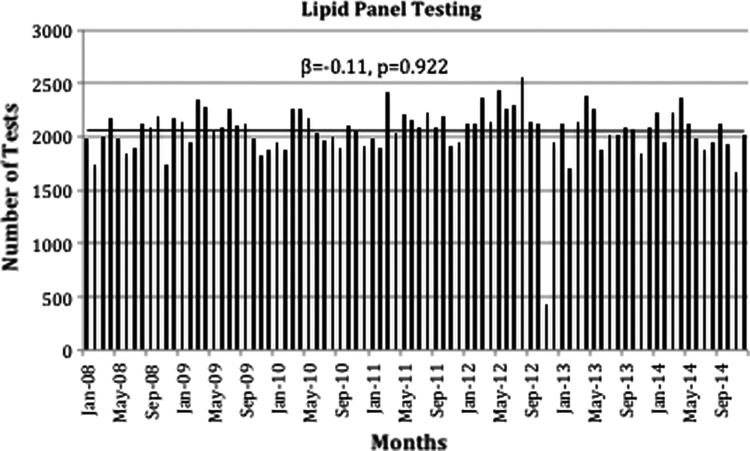
Lipid panel tests ordered from 2008 to 2014 by months. β, slope; p, *p*-value.

Subset analysis was also performed to further quantify the USPSTF guideline changes on screening with regards to age. Figure 3 demonstrates trends in PSA screening along the following age groups: <40, 40–54, 55–69, and ≥70. Decrease in screening were noted in all age groups except <40 years, which remained stable (β=0.61, *p*=0.730). The largest decline was noted in patients within the 55–69 age group, which also had the highest number of PSA screening tests (β=−173.32, *p*<0.001). Similar to the observed changes in PSA screening, we also observed a decline in prostate biopsies as shown in Figure 4 (β=−10.04, *p*=0.003). During this time period, we observed a 73% decrease in the rate of prostate biopsies.

**FIG. 3.**
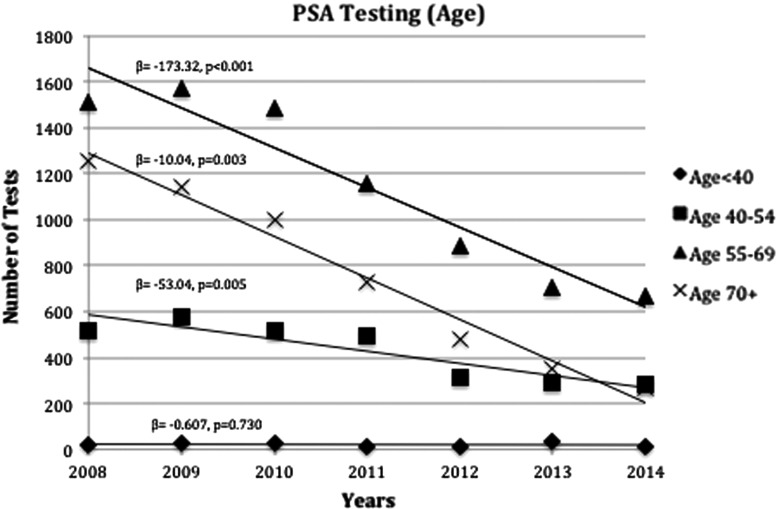
PSA tests ordered from 2008 to 2014 by age stratification. β, slope; p, *p*-value.

**FIG. 4.**
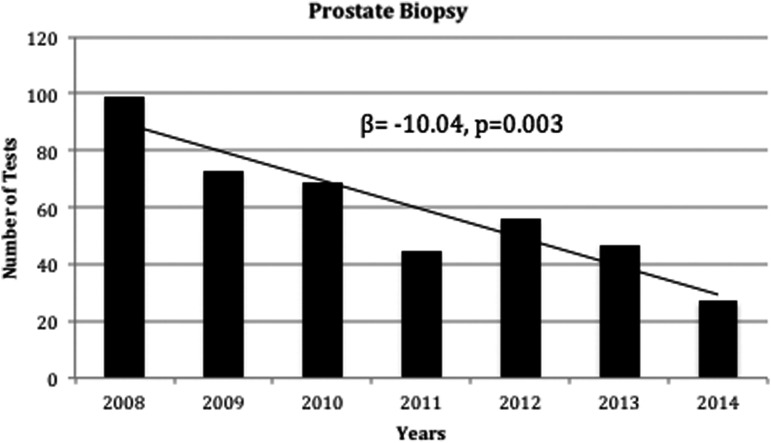
Prostate biopsies performed from 2008 to 2014. β, slope; p, *p*-value.

## Discussion

Less PSA screening may lead to missed opportunities for detection of cancer at an early stage and preventing death especially in groups at risk for aggressive prostate cancer (e.g., AA men). The USPSTF grade D recommendation against the screening of prostate cancer released in 2012 has led to major debates on how PSA screening should and should not be done, how to better diagnose prostate cancer, and determine which diagnosed cancers should be left untreated.^25–27^

Our investigation preceded the USPSTF report by 4 years and showed that a decline in PSA screening began 2 years before the USPSTF guidelines were published. In contrast, cholesterol screening with the use of lipid panel testing demonstrated no change during the study period suggesting that practitioners had maintained their interest in lipid panel testing while that in PSA testing declined. Our results are in agreement with Aslani et al., where they observed a decline in PSA screening from March 2009 to May 2012 (β=−0.001, *p*<0.001), which was continued thereafter with the release of the May 2012 USPSTF guidelines.^25^ The decrease that was most pronounced in the 55–69 age group occurring 2 years before the USPSTF guidelines may be related to the influence of the PCLO and ERSPC trials.^28^ This may reflect ongoing attitudes with PSA screening early on, and the decline noted in 2012 after the USPSTF guidelines could stem from the continued decline as a result of the PLCO and ERSPC trials, the 2012 USPSTF recommendation, a combination of the two, or other unknown factors. It is also possible that this change in attitude before the release of the guidelines may have exacerbated the decline.

This study also demonstrated a decrease in PSA screening in all age groups except 40 years and younger, with the largest decline in ages 55–69. Similarly, with other studies this trend extended to patients of all ages, especially from age 50 and above.^25,27,29,30^ These findings are in contrast to current AUA guidelines, in which PSA testing should be offered to patients age 55–69 after shared decision making. The decline seen may be evidence of variation in screening recommendations that preceded, but could be further influenced by the USPSTF recommendations. Our observations also highlight the importance of consensus in the use of any screening test among practitioners from different specialties who all wish to optimize the efficacy and safety of early detection.^31^

In our study, our rate of biopsies decreased during this time period. Prostate biopsies are generally safe and informative, but do have reported morbidity rates that include anxiety, infection, bleeding, pain, and sepsis.^32^ One of the perceived risks of PSA screening, therefore, has been requiring all men with an abnormal PSA to undergo the risks of prostate biopsy even though the majority of these men may have no cancer. Banerji et al. noted that a decrease in prostate biopsies is associated with a decrease in the detection of potentially curable so-called “intermediate” risk cancer.^33^ Bhindi et al. reviewed nearly 3400 prostate biopsies within the University Health Network of Toronto and also noted that PSA and prostate biopsy rates decreased due to USPSTF recommendations and that the median number of intermediate and high grade prostate cancers diagnosed per month decreased from 17.5 to 10.0 (*p*<0.001).^34^ Olsson et al. demonstrated that after the USPSTF guidelines, prostate biopsies were associated with an increase in higher risk features including a higher number of positive biopsy cores and a greater incidence of high grade disease.^35^ These findings suggest that a downside of decreased PSA screening is that patients may present with higher risk disease and that the possible benefits of reduced PSA screening could be mitigated by an increase in cancer-related morbidity and mortality.

The patient population of interest resides within a DOH designated medically underserved area. Furthermore, between 2008 and 2014, over 80% of our patients have AA or Hispanic backgrounds. This is in contrast to a rate of 13.3% AA and 17.8% Hispanic or Latino Americans residing in the United States, while over 70% are Caucasian.^36^ Despite trends and recommendations that may be appropriate for the general U.S. population, practitioners should be mindful when dealing with a different patient composition, especially one that is at higher risk for prostate cancer. The AA and African-Caribbean populations have shown to have up to 60% greater risk for prostate cancer compared to Caucasian counterparts.^37^ Multifactorial reasons may exist for the increased incidence due to race such as genomic changes related to prostate cancer, which are more common in these populations and increased testosterone levels compared to other races.^38,39^

Our study was unique in focusing on the trends in a cancer-screening test at an urban hospital in an underserved population where the risk of prostate cancer is on average higher than surrounding populations.^22–24^ Such patients may be more adversely affected by declining PSA screening than lower at-risk populations. Patients with an AA and African-Caribbean ancestry, low socioeconomic status, lack of health insurance coverage, unequal access to healthcare services, and less continuity with a primary care physician are all at risk of more advanced prostate cancer, trends that could worsen with declining rates of early diagnosis.^40^

Limitations of our study include a retrospective study design and limitation to a single institution in a highly populated demographic area. Validation with larger cohorts is needed. Comparison of PSA to lipid panel testing to provide for changes in patient volume is an adjustment that relies on providers and their practice patterns that could not be further evaluated or controlled. Similar to the study by Aslani et al., we believed that our approach to screening tests would take routine patient visits into account and be an appropriate measure of comparison.^25^

## Conclusion

Our findings demonstrate a statistically significant decrease in PSA screening and prostate biopsies within an urban population that preceded national USPSTF recommendations against PSA screening that are invariably due to other factors besides the 2012 guidelines. It is possible that the controversy surrounding the PSA debate resulted in physicians more carefully selecting patients for PSA screening. It is important that as we screen fewer patients for prostate cancer we develop strategies to continue making PSA screening available for those most at risk, minority men and those with difficult access to healthcare. Further studies are needed to determine whether there is any additional prostate cancer mortality because of decreased screening and prostate biopsies in these underserved populations and if any of these trends are reversed with the recent reversal in USPSTF recommendations.

## Abbreviations Used

AA: African Americans
ACS: American Cancer Society
AS: active surveillance
AUA: American Urological Association
DOH: Department of Health
ERSPC: European Randomized Study of Screening for Prostate Cancer
PLCO: Prostate, Lung Colorectal and Ovarian
PSA: Prostate Specific Antigen
USPSTF: United States Preventative Services Task Force
